# Sox9-expressing cells promote regeneration after radiation-induced lung injury via the PI3K/AKT pathway

**DOI:** 10.1186/s13287-021-02465-9

**Published:** 2021-07-02

**Authors:** Shuang Chen, Kang Li, Xinqi Zhong, Ganping Wang, Xiaocheng Wang, Maosheng Cheng, Jie Chen, Zhi Chen, Jianwen Chen, Caihua Zhang, Gan Xiong, Xiuyun Xu, Demeng Chen, Heping Li, Liang Peng

**Affiliations:** 1 Center for Translational Medicine, Institute of Precision Medicine, Department of Medical Oncology, The First Affiliated Hospital, Sun Yat-sen University, Guangzhou, 510080 China; 2 Department of Neonatology, The Third Affiliated Hospital of Guangzhou Medical University, Guangzhou, Guangdong China; 3 Department of Oral and Maxillofacial Surgery, Hospital of Stomatology, Guanghua School of Stomatology, Sun Yat-sen University, Guangzhou, 510030 China; 4 Oncology Department, Chinese PLA General Hospital, Beijing, 100000 China

## Abstract

**Background:**

Radiation-induced lung injury (RILI) is considered one of the most common complications of thoracic radiation. Recent studies have focused on stem cell properties to obtain ideal therapeutic effects, and Sox9 has been reported to be involved in stem cell induction and differentiation. However, whether Sox9-expressing cells play a role in radiation repair and regeneration remains unknown.

**Methods:**

We successfully obtained *Sox9*^CreER^, *Rosa*^tdTomato^ and *Rosa*^DTA^ mice and identified Sox9-expressing cells through lineage tracing. Then, we evaluated the effects of the ablation of Sox9-expressing cells in vivo. Furthermore, we investigated the underlying mechanism of Sox9-expressing cells during lung regeneration via an online single-cell RNA-seq dataset.

**Results:**

In our study, we demonstrated that Sox9-expressing cells promote the regeneration of lung tissues and that ablation of Sox9-expressing cells leads to severe phenotypes after radiation damage. In addition, analysis of an online scRNA-Seq dataset revealed that the PI3K/AKT pathway is enriched in Sox9-expressing cells during lung epithelium regeneration. Finally, the AKT inhibitor perifosine suppressed the regenerative effects of Sox9-expressing cells and the AKT pathway agonist promotes proliferation and differentiation.

**Conclusions:**

Taken together, the findings of our study suggest that Sox9-expressing cells may serve as a therapeutic target in lung tissue after RILI.

## Introduction

Approximately 4.3 million new cancer cases (thoracic cancer) and 2.9 million cancer-related deaths were reported in China alone in 2018, and most patients had unresectable disease [1]. Radiotherapy has been established as one of the most effective treatments for thoracic malignancies [2, 3]. However, this therapy inevitably causes adverse effects, as it can damage healthy tissues in the radiation field. It has been reported that 5–15% of patients suffer from radiation-induced lung injury (RILI) after thoracic radiation [4, 5]. In the clinic, RILI can develop into radiation pneumonia in the acute phase or into radiation-induced pulmonary fibrosis.

Recent studies have concentrated on the potential of stem cells for the treatment of lung-associated diseases, including RILI [6–8]. In addition, radiation resistance and potential regeneration capacity have been defined as traits of stem cells in mouse models [9]. Hence, stem cells seem to represent a feasible strategy to reduce radiation damage. However, the underlying molecular mechanisms modulating stem cell maintenance and regeneration during radiation are largely unknown. Significant contributions to new interventions for protecting against damage and promoting the repair of radiation-induced lung injury could be made after elucidating these genetic and cellular mechanisms.

Over the past decade, large strides have been made in the identification of stem cells and how they contribute to regeneration and repair. SOX9, a member of the sry-box-containing (SOX) family of proteins, was identified as a stem cell biomarker by lineage tracing in different organs [10, 11]. For instance, SOX9 is required for hair induction and directs outer root sheath cells to differentiate [12]. More importantly, SOX9 ablation combined with lineage tracing in the intestinal epithelium demonstrated that SOX9 could promote resistance to radiation in mouse intestinal stem cells [13].

Previous studies have shown that SOX9 plays a regenerative role in pulmonary epithelial cells [14, 15], showing that SOX9 may regulate proliferative ability after radiation injury. Nevertheless, whether Sox9-expressing cells function as stem cells in RILI and are endowed with radiation resistance and regeneration remains unclear.

In our study, we uncovered a previously undefined role of Sox9-expressing cells in repair and regeneration during RILI. Our results show that the number of Sox9-expressing cells was increased after radiation-induced lung damage and that Sox9-expressing cells were indispensable for repair and reconstruction. Single-cell RNA sequencing (scRNA-Seq) analysis showed that the expression level of genes in the PI3K/AKT pathway was increased in Sox9-overexpressing cells. The blockage of PI3K/AKT signalling using a small molecular inhibitor greatly repressed the regenerative ability of Sox9-expressing cells. Taken together, our data show that Sox9-expressing cells are an important factor in the repair and regeneration of radiation-induced lung injury.

## Materials and methods

### Mouse models

*Sox9*^CreER^, *Rosa*^tdTomato^ and *Rosa*^DTA^ mice were obtained from The Jackson Laboratory. For lineage tracing, *Sox9*^CreER^; *Rosa*^tdTomato^ mice were intraperitoneally injected with tamoxifen (Sigma, T5648-1G) at a dose of 0.08 mg/g body weight every day for three consecutive days. After radiation treatment, *Sox9*^CreER^, *Rosa*^tdTomato^ and *Rosa*^DTA^ mice were sacrificed at different time points (3, 7, 14 and 30 days). For PI3K/AKT inhibitor treatment, irradiated *Sox9*^CreER^; *Rosa*^tdTomato^ mice were assigned to the control and perifosine (250 mg/kg body weight per week, Beyotime, SC0227) treatment groups. For PI3K/AKT agonist treatment, irradiated *Sox9*^CreER^; *Rosa*^tdTomato^ mice were assigned to the control and SC79 (5 mg/kg body weight per week, Beyotime, SF2730) treatment groups. After 3, 7 and 14 days of treatment, lung samples were collected and injected with EdU 2 h prior to euthanasia. Both male and female mice were used, and all animal experiments were performed according to the Institutional Animal Care and Use Committee-approved protocols. For euthanasia, the mice were placed in new cages, euthanized immediately with 100% carbon dioxide and decapitated within 5 min for tissue collection.

### Mouse radiation experiments

Six- to 8-week-old C57BL/6 mice were first anaesthetized with intraperitoneal injection of 0.1 mL/20 g body weight ketamine/xylazine cocktail in the Animal Center of Sun Yat-sen University. The mice were then shielded with lead to protect the head, abdomen and extremities from radiation. Subsequently, the mice received whole-thorax radiation at a single dose of 16 Gy (165 MV, 25 mA X-rays; Rs2000, USA). The mice were randomly divided into two groups before radiation: the radiation group and the control group (3 mice in each group).

### Haematoxylin and eosin staining

Lung specimens were collected from the mice in the knockout and control groups and fixed in 4% paraformaldehyde (Biosharp, BL539A) for more than 24 h at 4 °C. After washing with PBS, the samples were dehydrated in gradient ethanol and embedded in paraffin. Then, paraffin sections (3–5 μm) of lung tissue were stained according to the standard protocol (Solarbio, G1120-100). In brief, the slides were stained with haematoxylin (2 min), differentiated with 1% hydrochloric acid ethanol (5 s), stained with eosin (1 min), dehydrated in ethanol and sealed with neutral balsam medium.

### Immunohistochemical staining and IHC score

For immunological analyses, 3–5-μm microtome sections were deparaffinized for 30 min and rehydrated with gradient alcohol. Then, the endogenous peroxidase activity of the sections was quenched with 3% H_2_O_2_, and heat-induced epitope retrieval was performed. The sections were incubated with primary antibodies targeting the following proteins at 4 °C overnight: SOX9 (1:200; Millipore, AB5535), Ki67 (1:500; Novus, NB500170), CD45 (1:200; Santa Cruz, sc-53665), α-SMA (1:200; CST,19245S), PDPN (1:200; abcam, ab256559), CC10 (1:200; Santa Cruz, sc-365992), Caspase-3 (1:200; CST, 9661S), SOX2 (1:300; abcam, ab97959), Oct3/4 (1:300; BD, BD611203), Klf4 (1:300; CST, 12173S) and cMyc (1:300; CST, 5605S). The specimens were washed with phosphate-buffered saline (PBS) three times prior to incubation with secondary antibodies targeting the primary antibodies for 30 min at 37 °C. For enzymatic assays, a horseradish peroxidase (HRP) conjugate was used for detection. The IHC score was calculated by multiplying the percentage of positive cells by the intensity. The intensity was scored as 0 (negative), 1+ (weak staining), 2+ (moderate staining) or 3+ (strong staining), while the frequency was scored according to the proportion of positive cells.

### Immunofluorescence staining

Tissues were fixed in 4% paraformaldehyde and treated with Triton X-100 (Sigma, USA) permeabilization buffer. Following treatment, lung specimens were blocked with PBS containing 1% bovine serum at room temperature and incubated with primary antibody at 4 °C. The samples were stained with DyLight 488 and Fluor 594 (Invitrogen, USA) for 1 h at room temperature, after which the cell nucleus was identified by DAPI staining at 1:1000 for 1 min. Images were obtained with a fluorescence microscope.

### Masson’s trichrome staining

The sections were dewaxed and rehydrated according to the process described above. To evaluate the degree of fibrosis and lesion localization, Masson’s trichrome staining was performed following the standard protocol (G1343, Solarbio).

### Single-cell analysis

scRNA-Seq data of mouse tracheal epithelial cells were analysed in this study. scRNA-Seq data from a total of 7662 cells (GEO number GSE102580) [16] were acquired from Gene Expression Omnibus (GEO, http://www.ncbi.nlm.nih.gov/geo/) database. The data were analysed using Seurat R package.

### Determination of NO and eNOs in vivo

Mouse serum concentrations of nitric oxide synthase 3, endothelial (eNOs), were determined by enzyme-linked immunosorbent assay (ELISA) kits (Elabscience Biotechnology, China). Mouse serum concentrations of nitric oxide (NO) were measured by nitric oxide (NO) assay kit (Jiancheng, China). Assays were performed according to the manufacturer’s protocols.

### Statistical analysis

All data were statistically analysed by GraphPad Prism 8.0. The data are expressed as the mean ± standard deviation (SD). Significant differences between groups were analysed by two-tailed unpaired Student’s t test. *P* < 0.05 was considered significant.

## Results

### Sox9-expressing cells contribute to the regeneration of the lung after radiation

**Fig. 1:**
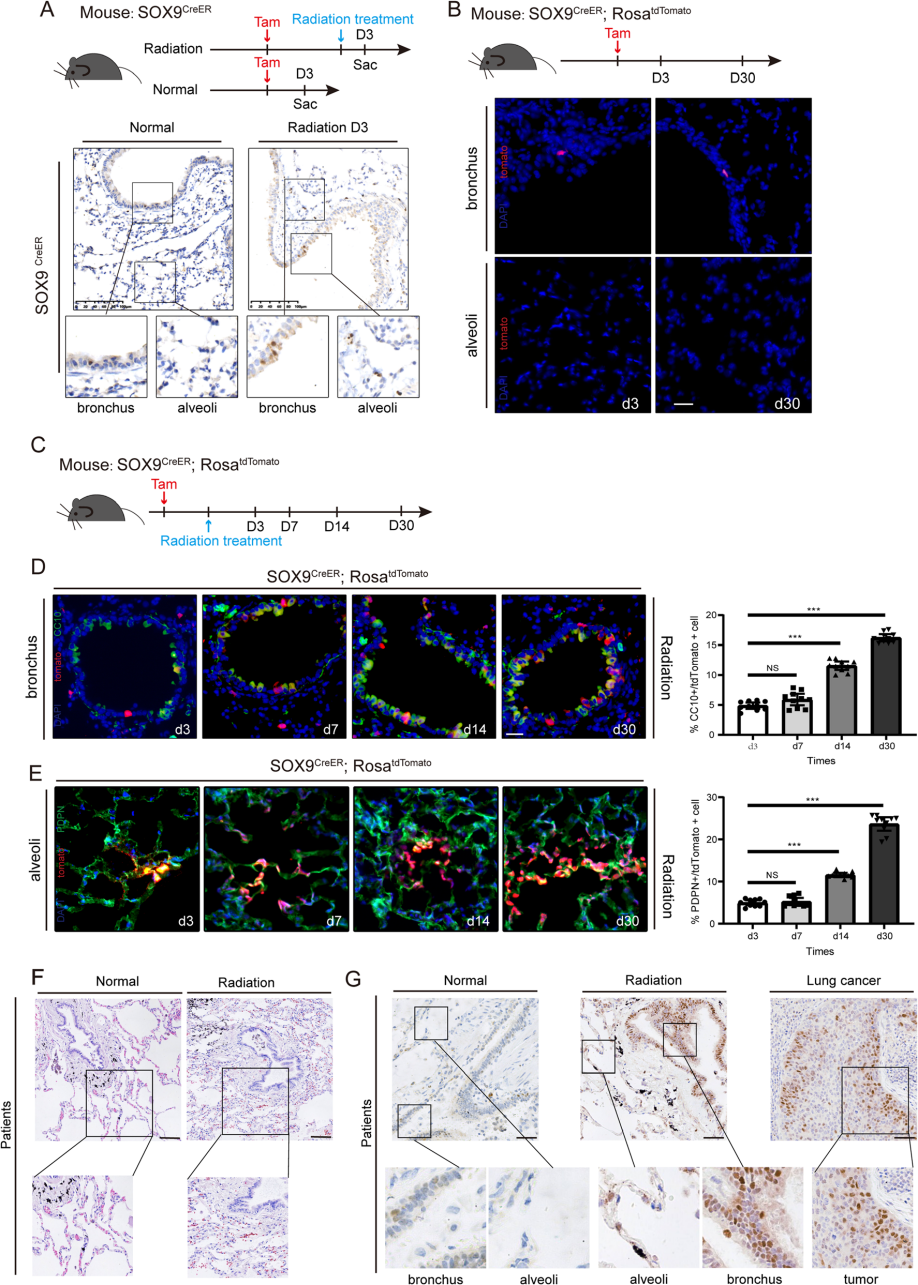
Sox9-expressing cells contribute to the regeneration of the lung after radiation. **A** Top: the experimental design for the control and radiation groups. Bottom: representative IHC staining images of Sox9 in the control group (left) and radiation damage group (right) of *Sox9*^CreER^ mice. Lung tissues were collected 3 days after radiation treatment. *n* = 3. Scale bar, 100 μm. **B** Top: the experimental design for Sox9^+^ cell lineage tracing for the control and radiation groups. Bottom: representative images of lineage tracing in lung tissue sections under homeostatic conditions. Nuclei were stained with DAPI (blue). Lung tissues were collected 3 and 30 days after radiation treatment. *n* = 3. Scale bar, 50 μm. **C** The experimental design for Sox9^+^ cell lineage tracing of *Sox9*^CreER^; *Rosa*^tdTomato^ mice after treatment with radiation. Before treatment, the mice were injected with tamoxifen (Tam). Mice were sacrificed 3, 7, 14 and 30 days after radiation treatment. *n* = 3. **D** Representative images of Sox9^+^ cell-driven lineage tracing in bronchi of *Sox9*^CreER^; *Rosa*^tdTomato^ mice after radiation injury (left). Percentage of CC10^+^/tdTomato^+^ cells was assessed (right). *p < 0.05, **p < 0.01 and ***p < 0.001 as determined by unpaired Student’s t test. Nuclei were stained with DAPI (blue). Lung tissues were collected 3, 7, 14 and 30 days after radiation treatment. *n* = 3. Scale bar, 50 μm. **E** Representative images of Sox9^+^ cell lineage tracing in the alveoli of *Sox9*^CreER^; *Rosa*^tdTomato^ mice after radiation injury. Percentage of PDPN^+^/tdTomato^+^ cells was assessed (right). *p < 0.05, **p < 0.01 and ***p < 0.001 as determined by unpaired Student’s t test. Nuclei were stained with DAPI (blue). Lung tissues were collected 3, 7, 14 and 30 days after radiation treatment. *n* = 3. Scale bar, 50 μm. **F** Representative images of H&E staining in the lung tissue of normal controls and radiation patients. Scale bar, 100 μm. **G** Representative IHC staining images of Sox9 in normal lung tissue (left), after lung tissue damaged by radiation (middle) and lung cancer tissue (right) in patients. Scale bar, 50 μm

To characterize the expression of Sox9 in adult mouse lung tissue, we first performed an IHC assay on normal lung tissues. We found that Sox9 protein was sparsely expressed in the bronchus and barely detectable in the alveoli of the adult mouse lung (Fig. 1A). However, we observed elevated levels of Sox9 protein in the bronchi 3 days after radiation (Fig. 1A). Surprisingly, we also noticed the expression of Sox9 in the alveolar region 3 days after radiation (Fig. 1A). To investigate the role of Sox9-expressing cells in lung homeostasis and the healing process after radiation, we carried out lineage tracing using *Sox9*^*creER*^*; Rosa*^*Tomato*^ transgenic mice (Fig. 1B). Consistent with the IHC results, our lineage tracing data showed that Tomato^+^ cells were located in the normal lung epithelium but not in the alveolar region (Fig. 1B). When we traced these cells for 30 days under normal conditions, we found that the number of Tomato^+^ cells was the same as that at 3 days (Fig. 1B), suggesting that Sox9-expressing cells in the bronchus have limited functions in the normal lung under conditions of homeostasis.

To test whether Sox9-expressing cells play a role in radiation-induced lung regeneration, *Sox9*^*creER*^*; Rosa*^*Tomato*^ mice were first administered tamoxifen and 15 Gy of radiation in the lung region (Fig. 1C). After radiation treatment, immunofluorescent staining results showed that the number of Sox9-expressing cells in both bronchial and alveolar cells was obviously increased (Fig. 1D, E). The number of Sox9-expressing cells in the bronchus gradually increased over time after radiation (Fig. 1D). Similarly, we observed that Tom^+^ cell colonies, which can differentiate into both PDPN-positive alveolar type I cells and PDPN-negative alveolar cells, expanded. Moreover, Sox9 is closely related to lung regeneration and lung cancer in patients. Normal alveolar septa and well-dilated alveolar cavities were observed in normal lung tissue (Fig. 1F). However, the alveoli were not expanded, and the alveolar septal structure was not obvious after injury (Fig. 1F). The data showed that SOX9 expression was upregulated in both radiated tissue and lung cancer tissue (Fig. 1G). Taken together, our data indicate that Sox9-expressing cells can contribute to lung regeneration after radiation.

### Sox9-expressing cells are essential for the regeneration of the lung after radiation

**Fig. 2:**
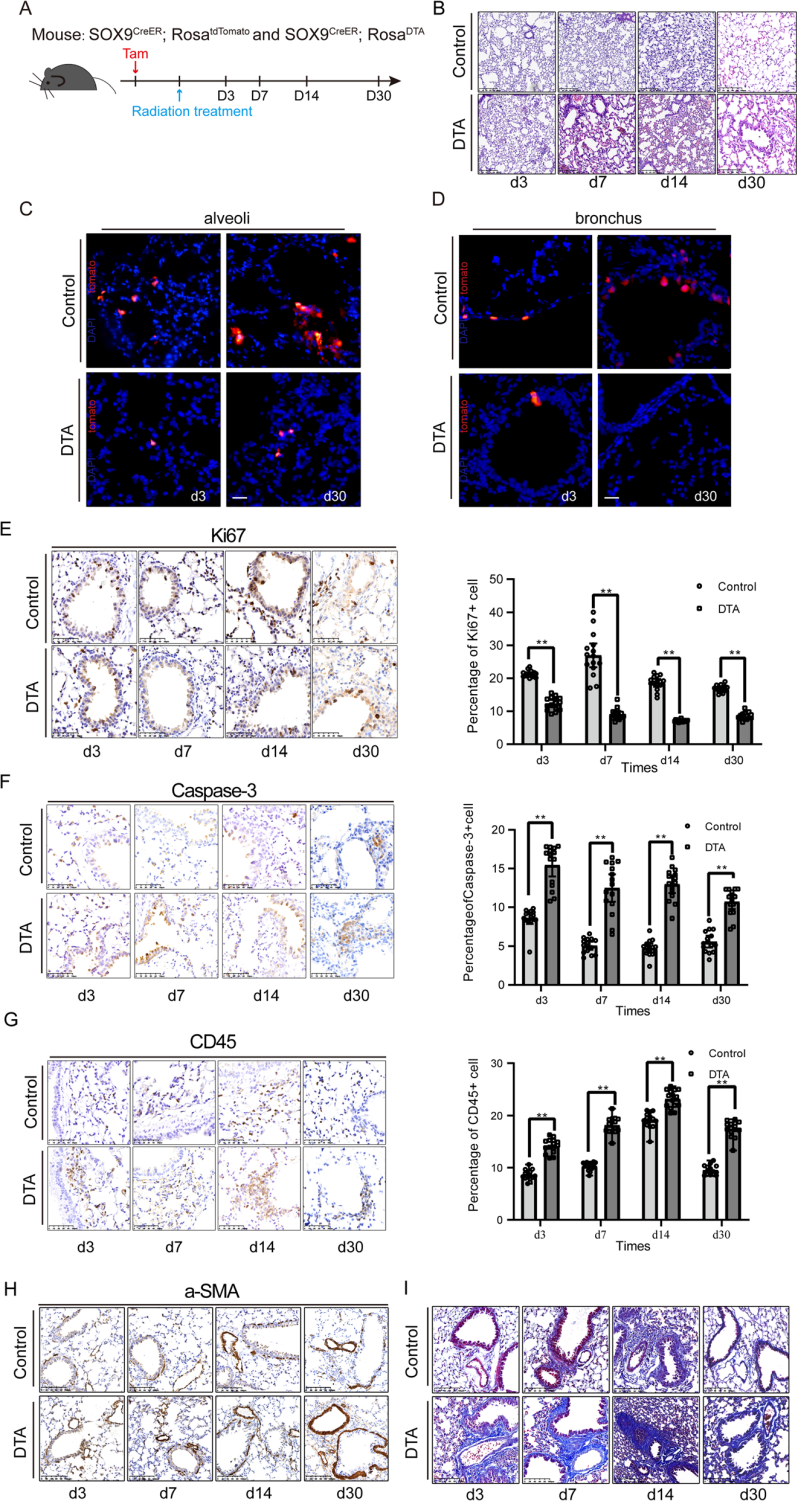
Sox9-expressing cells are essential for the regeneration of the lung after radiation. **A** The experimental design for Sox9-knockout and the control group lineage tracing after treatment with radiation (*n* = 4 per group). Before treatment, the mice were injected with tamoxifen (Tam). Mice were sacrificed 3, 7, 14 and 30 days after radiation treatment. *n* = 3. **B** Representative images of H&E staining of lung tissue section in Sox9-knockout and control mice. *n* = 3. Scale bar, 100 μm. **C**, **D** Immunostaining images of Sox9^+^ cell-driven lineage tracing in the alveoli and bronchus of Sox9-knockout and control mice. *n* = 3. Scale bar, 50 μm. **E**–**G** Representative IHC staining images of ki67, Caspase-3 and CD45 after radiation damage in *Sox9*^CreER^; *Rosa*^DTA^ mice and control mice (left). IHC staining scores of ki67, CD45 and Caspase-3 were assessed (right), respectively. *p < 0.05, **p < 0.01 and ***p < 0.001 as determined by unpaired Student’s t test. *n* = 3. Scale bar, 50 μm. **H** Immunostaining of a-SMA in *Sox9*^CreER^; *Rosa*^DTA^ mice and control mice. *n* = 3. Scale bar, 100 μm. **I** Representative images of Masson’s trichrome staining in *Sox9*^CreER^; *Rosa*^DTA^ mice and control mice. *n* = 3. Scale bar, 100 μm

To further investigate the specific role of Sox9-expressing cells during lung regeneration after radiation, we generated *Sox9*^*CreER*^*; Rosa*^*Tomato*^*; Rosa*^*DTA*^ mice that allow for tamoxifen-inducible expression of diphtheria toxin A (DTA), enabling the targeted ablation of Sox9-expressing cells (Fig. 2A). To examine the morphological changes in the lungs of mice after ablation of Sox9-expressing cells during radiation-induced regeneration, *Sox9*^*CreER*^*; Rosa*^*Tomato*^*; Rosa*^*DTA*^ mice and *Sox9*^*CreER*^*; Rosa*^*Tomato*^ transgenic littermates were subjected to histological examination. Our H&E staining results demonstrated that mouse lung tissue without Sox9-expressing cells displayed thickening of the alveolar septa and disruption of the integrity of pulmonary alveoli compared with the lung tissue of the control group after radiation (Fig. 2B), suggesting a loss of regenerative capacity. The lineage tracing results confirmed that the majority of Sox9-expressing cells were ablated after tamoxifen administration (Fig. 2C, D). Furthermore, ablation of Sox9-expressing cells led to a significant decrease in proliferating cells, increased apoptosis (Fig. 2E, F) and more severe lung inflammatory infiltration (Fig. 2G). Compared with the control groups, the group without Sox9-expressing cells showed aggravated radiation-induced pulmonary fibrosis (Fig. 2H, I). These data indicate the essential role of Sox9-expressing cells in the regeneration and repair of the lung after radiation in vivo.

### Sox9 maintains the regenerative ability of stem cells via the AKT/PI3K pathway

**Fig. 3:**
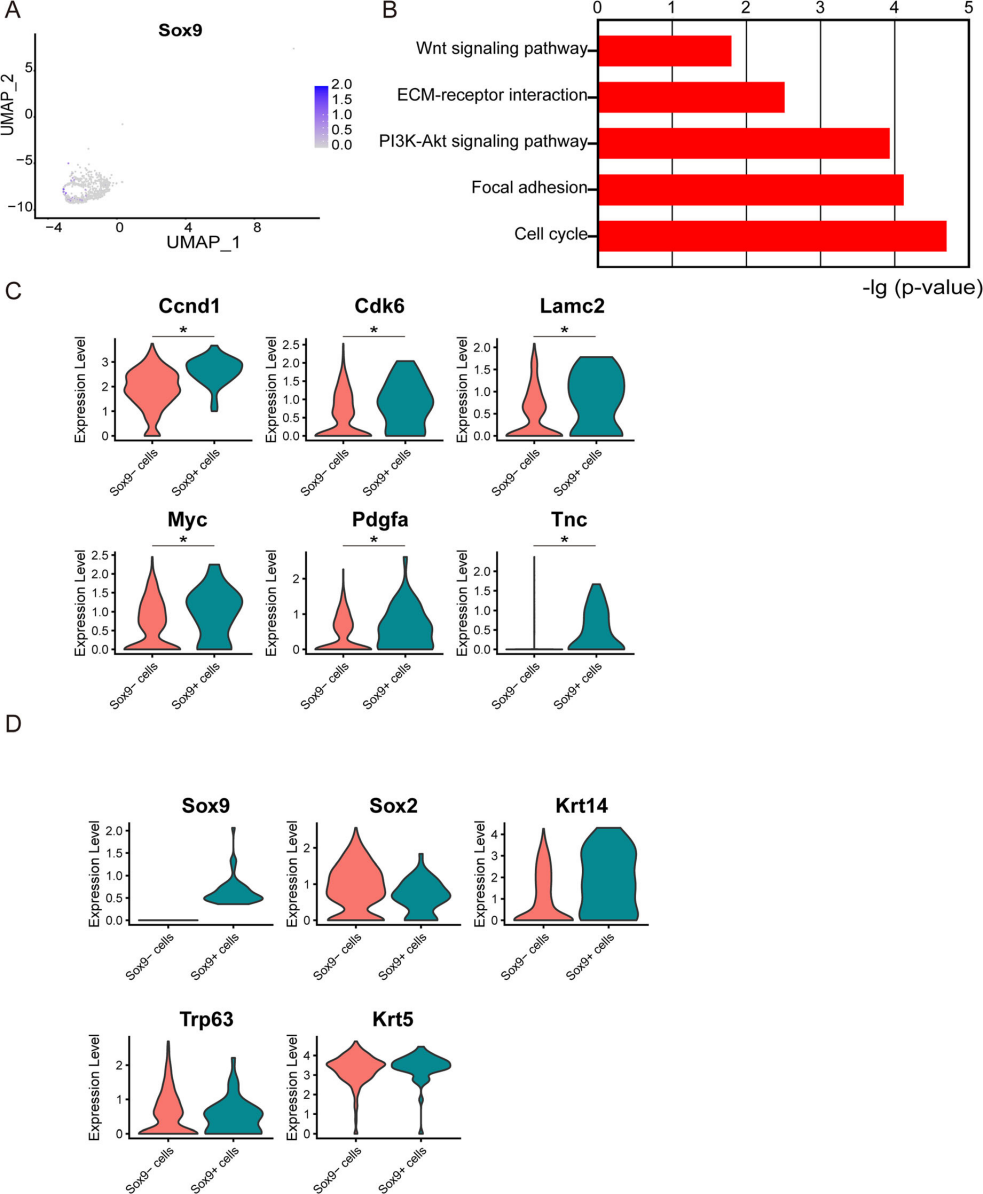
Sox9 maintains the regenerative ability of stem cells via the AKT/PI3K pathway. **A** Dot plots showing the pattern of Sox9 expression for the cell cluster on the UMAP map. **B** Enrichment of different KEGG pathways identified in the Sox9^+^ cell population in the scRNA-Seq data. **C** Violin plots depicting the expression of the indicated genes downstream of PI3K/Akt genes in Sox9+ and Sox9− cells. *p < 0.05, **p < 0.01 and ***p < 0.001 as determined by unpaired Student’s t test. **D** Violin plots depicting the expression of the indicated known stem cell markers in Sox9+ and Sox9− cells

To further investigate the underlying mechanism of Sox9-expressing cells during lung regeneration, we analysed an online single-cell RNA-seq dataset that characterized the cellular composition of regenerated mouse lung epithelium after polidocanol-induced injury (GSE102580) [16]. We first examined the expression of Sox9 in cycling basal keratinocytes, which are responsible for lung epithelium regeneration. In accordance with our above findings, we found that Sox9 was upregulated in a subset of cycling basal keratinocytes in the online dataset (Fig. 3A). We then divided the cycling basal keratinocytes into Sox9^+^ and Sox9^−^ populations. We used the Seurat R package to identify the differentially expressed genes between these two populations using a Wilcoxon rank sum test. We found that 117 genes were significantly increased in the Sox9^+^ population. KEGG pathway analysis of these genes identified enriched pathways, including the cell cycle, focal adhesion, PI3K-Akt signalling, ECM-receptor interaction and Wnt signalling pathways (Fig. 3B). Indeed, we found that downstream targets of the PI3K-Akt signalling pathway, such as Ccnd1, Cdk6, Lamc2, Myc, Pdgfa and Tnc, were dramatically downregulated (Fig. 3C). We then examined whether Sox9-expressing cells coexpressed previously known lung epithelium stem cell markers. Surprisingly, we detected no difference in the expression of Sox2, Krt14, Krt5 and Trp63 between the Sox9^+^ and Sox9^−^ populations (Fig. 3D), suggesting that Sox9-expressing cells did not overlap with previously identified lung epithelium stem cell populations.

### AKT/PI3K pathway inhibitor could attenuate the effect

**Fig. 4:**
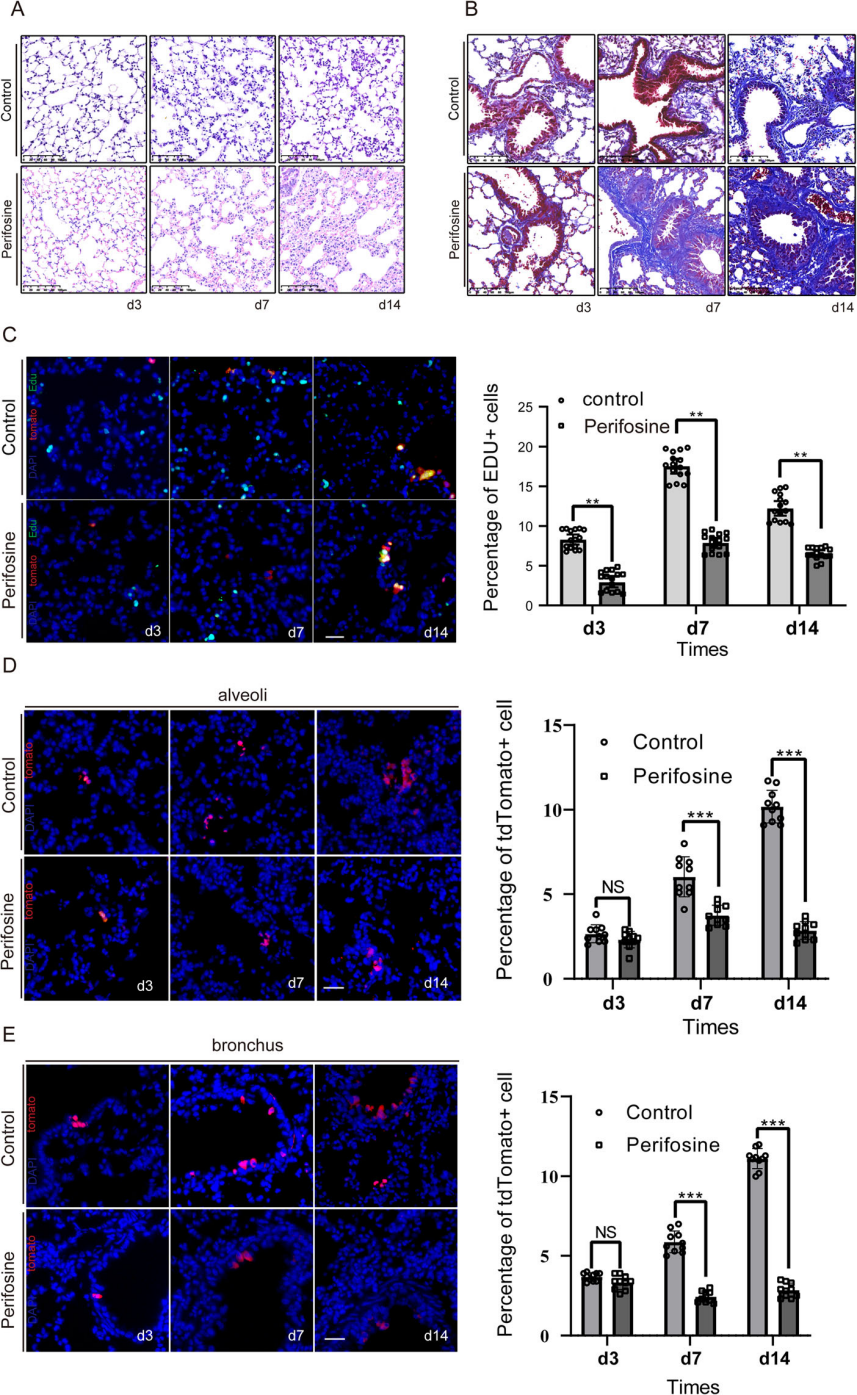
AKT/PI3K pathway inhibitor attenuated the effect. **A** Representative images of H&E staining of lung tissue sections from *Sox9*^CreER^; *Rosa*^tdTomato^ mice after RILI. An AKT inhibitor was intragastrically administered after radiation damage. Lung tissues were collected 3, 7 and 14 days after drug treatment. *n* = 3. Scale bar, 100 μm. **B** Representative images of Masson’s trichrome staining in the AKT inhibitor group and control group. Scale bar, 100 μm. **C** Immunostaining of EDU+ in *Sox9*^CreER^; *Rosa*^tdTomato^ mice after RILI and inhibitor treatment. The EDU+ staining score was assessed (right). *p < 0.05, **p < 0.01 and ***p < 0.001 as determined by unpaired Student’s t test. Scale bar, 50 μm. **D**, **E** Representative images of SOX9^+^ cell-driven lineage tracing in mice after radiation injury and inhibitor treatment. Percentage of tdTomato^+^ cells was assessed (right), respectively. *p < 0.05, **p < 0.01 and ***p < 0.001 as determined by unpaired Student’s t test. Nuclei are labelled with DAPI (blue). *n* = 3. Scale bar, 50 μm

Finally, to probe the possible functional role of the PI3K-AKT signalling pathway in regulating Sox9-expressing cells during radiation-induced lung regeneration, we used perifosine, a specific AKT inhibitor, to treat mice after radiation. As shown in Fig. 4A and B, after treatment with perifosine, the alveolar septum was thickened, and pulmonary fibrosis worsened. Similarly, the proliferation of pulmonary epithelial cells was significantly reduced (Fig. 4C). Lineage tracing results showed that Sox9-expressing cells were incapable of differentiating into functional cells, compared with the control group (Fig. 4D, E). Therefore, our data indicated that the regeneration and repair of Sox9-expressing cells after radiation injury is controlled by the PI3K/AKT signalling pathway.

### AKT/PI3K pathway agonist promotes proliferation and differentiation

**Fig. 5:**
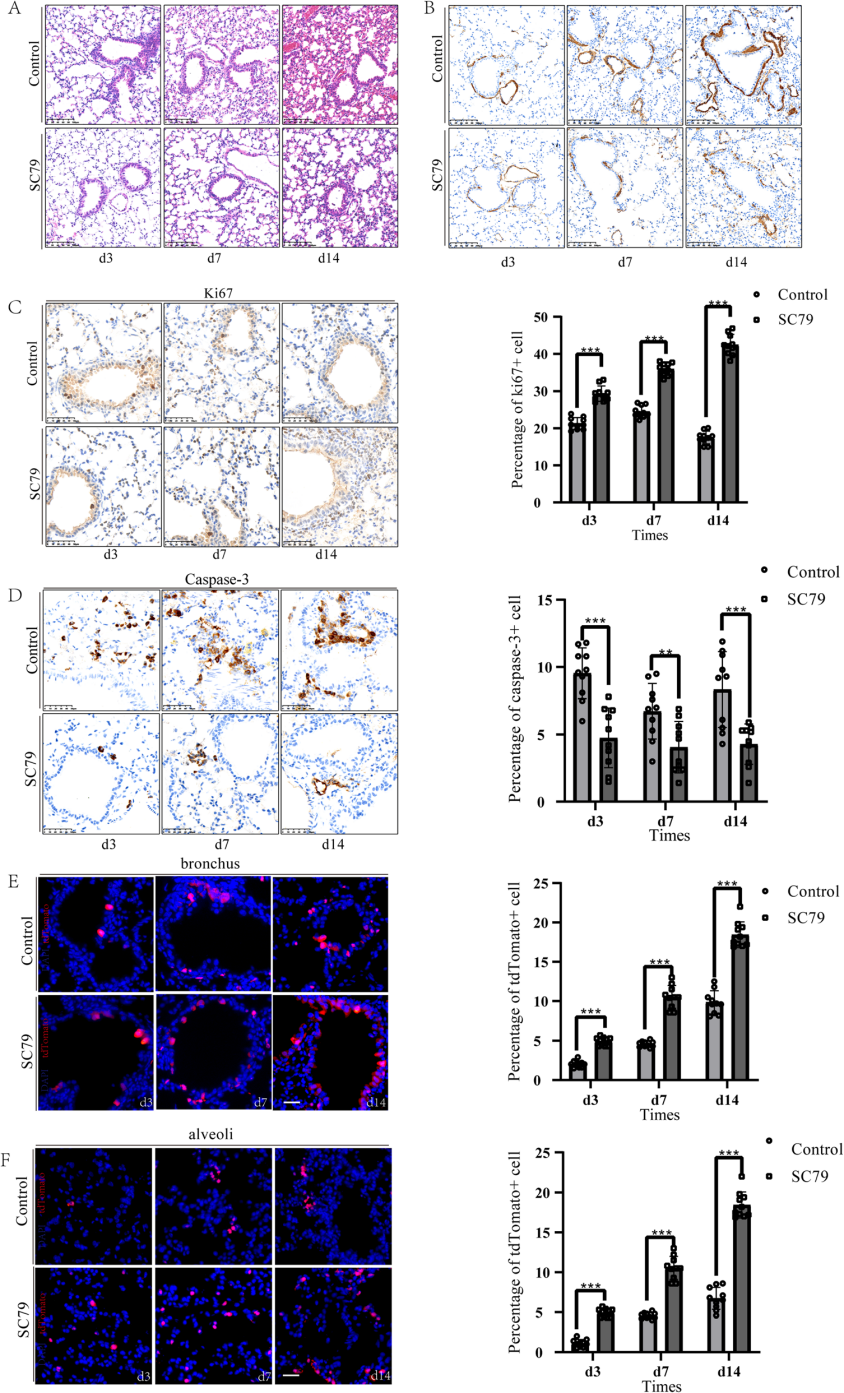
AKT/PI3K pathway agonist promotes proliferation and differentiation. **A** Representative images of H&E staining of lung tissue sections from *Sox9*^CreER^; *Rosa*^tdTomato^ mice after RILI. An AKT agonist was intragastrically administered after radiation damage. Lung tissues were collected 3, 7 and 14 days after drug treatment. *n* = 5. Scale bar, 100 μm. **B** Immunostaining of a-SMA in mice receiving SC79 group and control group. *n* = 5. Scale bar, 100 μm. **C**, **D** Representative IHC staining images of ki67 and Caspase-3 after radiation damage and agonist treatment (left). Percentage of ki67+ and Caspase-3+ cells were assessed (right), respectively. **p < 0.01 and ***p < 0.001 as determined by unpaired Student’s t test. *n* = 5. Scale bar, 50 μm. **E**, **F** Representative images of SOX9^+^ cell-driven lineage tracing in mice after radiation injury and agonist treatment (left). Percentage of tdTomato^+^ cells was assessed (right), respectively. *p < 0.05, **p < 0.01 and ***p < 0.001 as determined by unpaired Student’s t test. Nuclei are labelled with DAPI (blue). *n* = 5. Scale bar, 50 μm

Our data have indicated that PI3K-AKT signalling pathway was involved in Sox9-expressing cell stemness regulation. As we expected, AKT inhibitor perifosine can significantly weaken the regeneration and repair of Sox9-expressing cells after radiation injury. In comparison, with SC79 treatment, an AKT agonist, the alveolar septum was narrowed, and pulmonary fibrosis was improved (Fig. 5A, B). With SC79 stimulation, pulmonary epithelial cells show increased proliferation capacity and decreased apoptosis (Fig. 5C, D). Notably, in the lineage tracing study, the activation of AKT downstream signalling induced by SC79 endowed Sox9-expressing cells greater ability to differentiate into functional cells (Fig. 5E, F). These data collectively indicated SC79 promoted the proliferation and differentiation of Sox9-expressing cells through the activation of the Akt signalling pathway, which ultimately leads to RILF regeneration and repair.

### Stem cell markers were involved in RILI

**Fig. 6:**
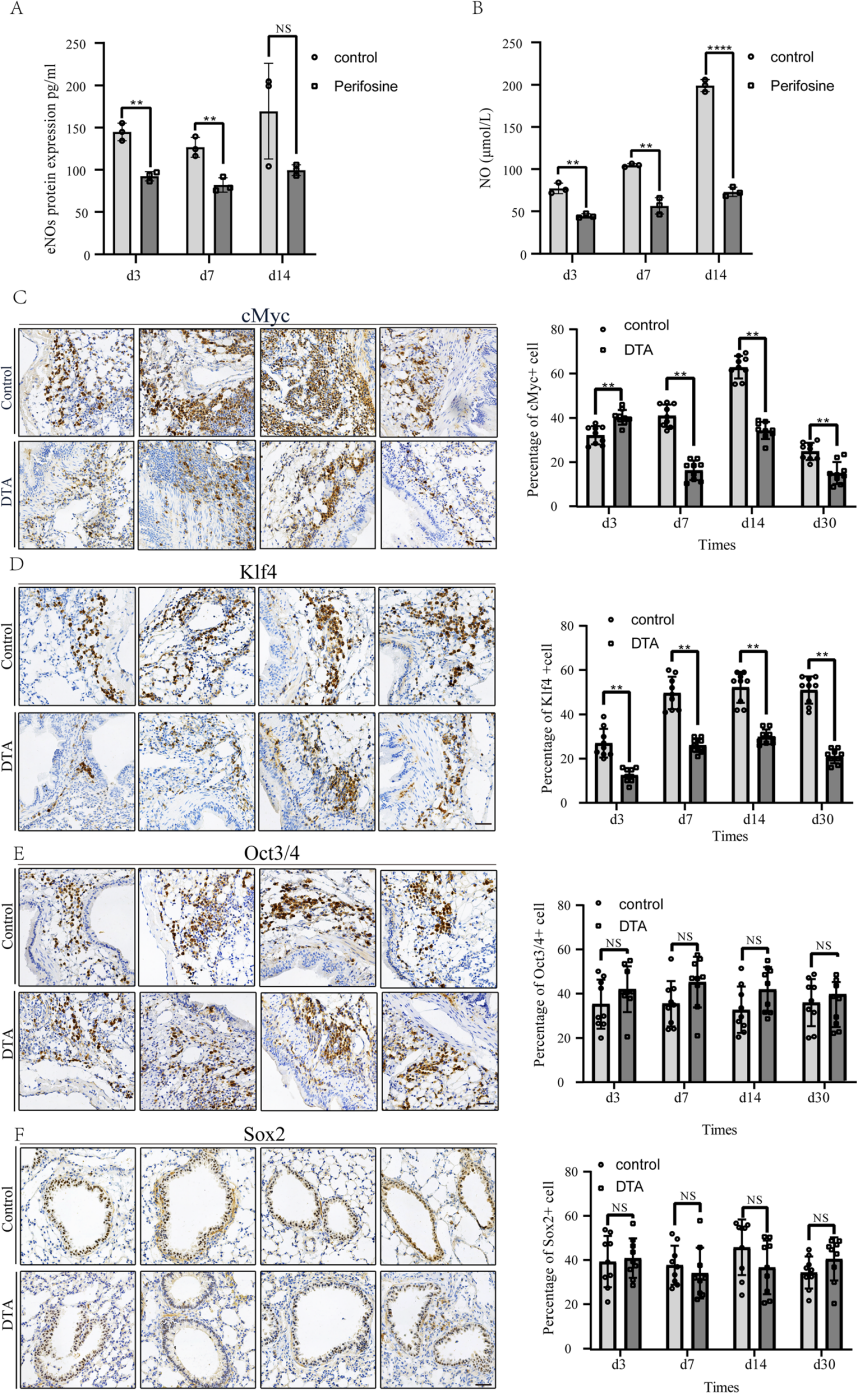
Stem cell markers were involved in RILI. **A** Nitric oxide synthase 3, endothelial (eNOs) level in mouse serum. Serums were collected after 3, 7 and 14 days after radiation treatment. *n* = 3. **B** Nitric oxide (NO) level in mouse serum. Serums were collected after 3, 7 and 14 days after radiation treatment. *n* = 3. **C**–**F** Representative IHC staining images of cMyc, Klf4, Oct3/4 and Sox2 after radiation damage in SOX9^CreER^; Rosa^DTA^ mice and control mice (left). The IHC staining scores of cMyc, Klf4, Oct3/4 and Sox2 were assessed (right). *p < 0.05, **p < 0.01, ***p < 0.001 and ***p < 0.0001 as determined by unpaired Student’s t test. *n* = 3. Scale bar, 50 μm

Based on previous research and our results, we decided to further analyse how the PI3K-AKT signalling pathway was involved. To date, NO simulates cell proliferation and was driven by the AKT signalling. Interestingly, we observed the levels of NO and eNOs were highest after 14 days of radiation, indicating activation of the AKT-mediated eNOs and NO pathway. Compared with the control group, eNOs and NO levels were decreased in all perifosine treatment groups, showing the pathway was restrained (Fig. 6A, B).

A large number of studies have shown that stem cell markers play a key role in radiation injury regeneration. To investigate whether other stem cell markers are involved in RILI repair, we performed a series of staining assays. After the ablation of Sox9-expressing cells, the expression of cMyc and Klf4 was significantly decreased (Fig. 6C, D). However, the expression of Oct3/4 and Sox2 did not differ from that in the control groups (Fig. 6E, F), which was consistent with previous data. Based on this result, we believe that stem cell markers are closely involved in RILI regeneration and repair.

## Discussion

RILI, including radiation pneumonia and radiation-induced pulmonary fibrosis, is characterized by an inflammatory phenotype and cell repair disorder [17]. Notably, emerging evidence emphasizes the role of stem cells in RILI, providing a new perspective for obtaining better treatment efficacy. In our study, the data showed the profound role of Sox9-expressing cells in repair and regeneration in response to irradiation via the PI3K/AKT signalling pathway.

Recent studies focused on stem cells have shed light on how to utilize unique molecular markers to identify cells with regenerative ability [18, 19]. Experimental analyses, such as transcriptomics, proteomics and lineage tracing, have proven that many biomarkers characterize a certain stem cell population [20–22]. Data showed that Sox9-expressing cells are rare, but they are required for regeneration after RILI. In addition to influencing lung cell proliferation, we found that alveolar epithelial cells became radiosensitive after Sox9-expressing cells were ablated. Moreover, Sox9-expressing cell ablation resulted in more severe lesions in lung tissue with higher levels of inflammation and more extensive fibrosis. Notably, although our data verified that Sox9-expressing cells play a key role in irradiation resistance, the specific mechanism by which Sox9-expressing cells participate needs further analysis.

We found that Sox9-expressing cells are endowed with a regenerative phenotype after RILI. Subsequently, the specific mechanism by which Sox9-expressing cells participate in cell proliferation was analysed by scRNA-Seq data. Notably, the upregulated genes in Sox9-expressing cells were significantly enriched in the PI3K/AKT signalling pathway, which is one of the most frequently activated signalling pathways in cancers, showing that SOX9 may have unknown functions relevant to oncology therapy that merit further investigation. Previous studies have shown that repressing the PI3K/AKT signalling pathway predisposes cells to reduced DNA repair capacity, resulting in an increase in radiation-induced apoptosis [23]. Consistent with a radioresistant phenotype, another study demonstrated that PI3K/AKT-dependent pathways are protective or promote regeneration after radiation injury [24]. Recent cell signalling studies showed that SOX9 could activate the PI3K/AKT signalling pathway and promote cellular proliferation [25, 26], both of which indicate the potential role of SOX9 in regeneration and repair. Furthermore, the in vivo AKT inhibitor and agonist assay results further confirmed our hypothesis; inactivation of the AKT pathway has been shown to decrease cellular proliferation, and Sox9-expressing cells fail to differentiate while AKT pathway activation yielded the opposite result. Taken together, the results of these studies support the explanation that Sox9-expressing cells are involved in regeneration and repair by activating the PI3K/AKT signalling pathway after RILI, indicating that SOX9 could be a potential therapeutic target to promote regeneration and repair in patients with thoracic malignancies.

## Conclusion

In summary, our study provides a model in which Sox9-expressing cells serve as a functional cell population in pulmonary epithelial cells, imparting regenerative and repair capacities to stem cells by activating the PI3K/AKT pathway. Despite the fact that radiation-induced lung injury, one of the common injuries caused by chest radiotherapy, has presented challenges to patients’ quality of life and disease prognosis for a long time, there may be approaches to mediate these effects.

## Data Availability

The data used to support the findings of this study are available from the corresponding authors upon request.

## Abbreviations

RILI: Radiation-induced lung injury
SOX: A member of sry-box-containing
scRNA-Seq: Single-cell RNA sequencing
PBS: Phosphate-buffered saline
HRP: Horseradish peroxidase
GEO: Gene Expression Omnibus
SD: Standard deviation
DTA: Diphtheria toxin A
Tam: Tamoxifen
eNOs: Nitric oxide synthase 3, endothelial
NO: Nitric oxide
ELISA: Enzyme-linked immunosorbent assay

## References

[1] Feng RM, Zong YN, Cao SM, Xu RH (2019). Current cancer situation in China: good or bad news from the 2018 Global Cancer Statistics?. Cancer Commun (Lond).

[2] Bradley JA, Mendenhall NP (2018). Novel radiotherapy techniques for breast cancer. ANNU REV MED.

[3] Ko EC, Raben D, Formenti SC (2018). The integration of radiotherapy with immunotherapy for the treatment of non-small cell lung cancer. CLIN CANCER RES.

[4] Giuranno L, Ient J, De Ruysscher D, Vooijs MA (2019). Radiation-induced lung injury (RILI). FRONT ONCOL.

[5] Hanania AN, Mainwaring W, Ghebre YT, Hanania NA, Ludwig M (2019). Radiation-induced lung injury: assessment and management. CHEST.

[6] Jones-Freeman B, Starkey MR (2020). Bronchioalveolar stem cells in lung repair, regeneration and disease. J PATHOL.

[7] Mobius MA, Thebaud B (2016). Cell therapy for bronchopulmonary dysplasia: promises and perils. PAEDIATR RESPIR REV.

[8] Zanoni M, Cortesi M, Zamagni A, Tesei A. The Role of Mesenchymal Stem Cells in Radiation-Induced Lung Fibrosis. Int J Mol Sci. 2019;20(16):3876. 10.3390/ijms20163876. 10.3390/ijms20163876 PMC6719901 31398940

[9] Barker N (2014). Adult intestinal stem cells: critical drivers of epithelial homeostasis and regeneration. Nat Rev Mol Cell Biol.

[10] Domenici G, Aurrekoetxea-Rodriguez I, Simoes BM, Rabano M, Lee SY, Millan JS, Comaills V, Oliemuller E, Lopez-Ruiz JA, Zabalza I (2019). A Sox2-Sox9 signalling axis maintains human breast luminal progenitor and breast cancer stem cells. ONCOGENE.

[11] Wang L, Zhang Z, Yu X, Huang X, Liu Z, Chai Y, Yang L, Wang Q, Li M, Zhao J, Hou J, Li F (2019). Unbalanced YAP-SOX9 circuit drives stemness and malignant progression in esophageal squamous cell carcinoma. ONCOGENE.

[12] Vidal VP, Chaboissier MC, Lutzkendorf S, Cotsarelis G, Mill P, Hui CC, Ortonne N, Ortonne JP, Schedl A (2005). Sox9 is essential for outer root sheath differentiation and the formation of the hair stem cell compartment. CURR BIOL.

[13] Roche KC, Gracz AD, Liu XF, Newton V, Akiyama H, Magness ST (2015). SOX9 maintains reserve stem cells and preserves radioresistance in mouse small intestine. GASTROENTEROLOGY.

[14] Laughney AM, Hu J, Campbell NR, Bakhoum SF, Setty M, Lavallee VP, Xie Y, Masilionis I, Carr AJ, Kottapalli S (2020). Regenerative lineages and immune-mediated pruning in lung cancer metastasis. NAT MED.

[15] Nichane M, Javed A, Sivakamasundari V, Ganesan M, Ang LT, Kraus P, Lufkin T, Loh KM, Lim B (2017). Isolation and 3D expansion of multipotent Sox9(+) mouse lung progenitors. NAT METHODS.

[16] Plasschaert LW, Zilionis R, Choo-Wing R, Savova V, Knehr J, Roma G, Klein AM, Jaffe AB (2018). A single-cell atlas of the airway epithelium reveals the CFTR-rich pulmonary ionocyte. NATURE.

[17] Lu L, Sun C, Su Q, Wang Y, Li J, Guo Z, Chen L, Zhang H (2019). Radiation-induced lung injury: latest molecular developments, therapeutic approaches, and clinical guidance. CLIN EXP MED.

[18] Hatina J, Parmar HS, Kripnerova M, Hepburn A, Heer R (2018). Urothelial carcinoma stem cells: current concepts, controversies, and methods. Methods Mol Biol.

[19] Yu QC, Song W, Wang D, Zeng YA (2016). Identification of blood vascular endothelial stem cells by the expression of protein C receptor. CELL RES.

[20] Munoz J, Stange DE, Schepers AG, van de Wetering M, Koo BK, Itzkovitz S, Volckmann R, Kung KS, Koster J, Radulescu S (2012). The Lgr5 intestinal stem cell signature: robust expression of proposed quiescent ‘+4’ cell markers. EMBO J.

[21] Ragelle H, Naba A, Larson BL, Zhou F, Prijic M, Whittaker CA, Del RA, Langer R, Hynes RO, Anderson DG (2017). Comprehensive proteomic characterization of stem cell-derived extracellular matrices. BIOMATERIALS.

[22] Yan KS, Gevaert O, Zheng G, Anchang B, Probert CS, Larkin KA, Davies PS, Cheng ZF, Kaddis JS, Han A (2017). Intestinal enteroendocrine lineage cells possess homeostatic and injury-inducible stem cell activity. CELL STEM CELL.

[23] Liao J, Jin H, Li S, Xu L, Peng Z, Wei G, Long J, Guo Y, Kuang M, Zhou Q, Peng S (2019). Apatinib potentiates irradiation effect via suppressing PI3K/AKT signaling pathway in hepatocellular carcinoma. J Exp Clin Cancer Res.

[24] Yang L, Wang R, Gao Y, Xu X, Fu K, Wang S, Li Y, Peng R (2014). The protective role of interleukin-11 against neutron radiation injury in mouse intestines via MEK/ERK and PI3K/Akt dependent pathways. Dig Dis Sci.

[25] Chang L, Graham PH, Ni J, Hao J, Bucci J, Cozzi PJ, Li Y (2015). Targeting PI3K/Akt/mTOR signaling pathway in the treatment of prostate cancer radioresistance. Crit Rev Oncol Hematol.

[26] Wang L, Zhang Z, Yu X, Li Q, Wang Q, Chang A, et al. SOX9/miR-203a axis drives PI3K/AKT signaling to promote esophageal cancer progression. Cancer Lett. 2020;468:14–26. 10.1016/j.canlet.2019.10.004. 10.1016/j.canlet.2019.10.004 31600529

